# Predictors of time to feeding-tube independence in locally advanced head and neck cancer

**DOI:** 10.3389/fonc.2026.1956034

**Published:** 2026-10-20

**Authors:** Saeed Salman, Nancy Heppell, Nadin Dabbah Salman, Natali Shirron, Acheem Joseph, Christina Tsien, George Shenouda

**Affiliations:** 1 Division of Radiation Oncology, McGill University Health Centre, Montreal, QC, Canada; 2 Division of Radiation Oncology, Joseph Fishman Oncology Center, Rambam Health Care Campus, Haifa, Israel; 3 Department of Ophthalmology, Carmel Medical Center, Haifa, Israel

**Keywords:** chemoradiotherapy, dosimetric predictors, dysphagia, feeding tube, gastrostomy, head and neck cancer, overall survival, smoking

## Abstract

**Background:**

Prophylactic gastrostomy is widely used during definitive chemoradiotherapy for locally advanced head and neck cancer (LAHNC), yet some patients remain tube-dependent long after treatment. Whether prolonged dependence carries prognostic weight of its own, or simply mirrors tumour burden and performance status, is unclear. We examined predictors of time to feeding-tube independence and the association between persistent dependence and overall survival (OS).

**Methods:**

We reviewed 213 consecutive patients with LAHNC treated at a single institution (2015–2022) with definitive chemoradiotherapy to 70 Gy and prophylactic PEG/RIG placement. Nine patients with an inconsistent extraction date were excluded, leaving 204. Time to independence was analysed with Kaplan–Meier estimates and cause-specific multivariable Cox regression, treating death as a competing risk, with a Fine–Gray sensitivity model. Dosimetric variables and baseline Karnofsky performance status (KPS) were modelled continuously. To avoid immortal-time bias, OS was assessed with landmark analyses at 3, 6 and 12 months. Patients were classified by tube status at each landmark, with adjustment for KPS, T and N classification, smoking history and age.

**Results:**

Median time to independence was 3.2 months. Any smoking history predicted slower independence (HR 0.60, 95% CI 0.42–0.84; p=0.004), and higher KPS predicted faster independence (HR 1.02 per point, 95% CI 1.01–1.04; p=0.006). Higher middle pharyngeal constrictor dose was associated with slower recovery (HR 0.98 per Gy, 95% CI 0.96–1.00; p=0.048), and oral cavity dose showed a similar trend (p=0.067). In unadjusted landmark analyses, patients still dependent had worse OS at 6 months (HR 1.78, 95% CI 1.01–3.13) and 12 months (HR 2.53, 95% CI 1.30–4.91). After adjustment, the association was attenuated and non-significant at 3 months (aHR 1.35, 95% CI 0.76–2.42) and 6 months (aHR 1.48, 95% CI 0.81–2.70), but persisted at 12 months (aHR 2.20, 95% CI 1.05–4.58; p=0.036).

**Conclusions:**

Early tube dependence largely reflects baseline disease burden and performance status. Dependence persisting to one year, however, identifies patients with worse survival that these factors do not explain, and they may merit closer follow-up. Smoking history and middle constrictor dose are potentially modifiable contributors to slower recovery, supporting structured smoking-cessation care and dysphagia-optimised planning. Prospective validation is warranted.

## 1 Introduction

Concurrent chemoradiotherapy delivered to 70 Gy is the standard of care for locally advanced head and neck cancer (LAHNC) and yields durable locoregional control at the cost of substantial mucosal and swallowing toxicity. Pre-treatment malnutrition is reported in 35–60% of patients and is compounded during therapy by mucositis, xerostomia, odynophagia and dysgeusia, with critical weight loss linked to treatment interruption, immunosuppression, and reduced disease-specific survival (1–4).

Enteral nutritional support — most often via percutaneous endoscopic gastrostomy (PEG) or radiologically inserted gastrostomy (RIG) — has become a cornerstone of supportive care, deployed either prophylactically or reactively (5, 6). Although prophylactic placement reliably attenuates weight loss and reduces unplanned admissions, multiple large series, have failed to demonstrate a corresponding overall-survival benefit (7). In parallel, prolonged tube dependence has emerged as a clinically distinct entity associated with persistent dysphagia, aspiration, impaired quality of life, and — in at least two cohort studies — inferior overall survival (8, 9). These observations have shifted the central clinical question from whether to place a tube to which patients become tube-dependent in the long term, and which determinants are amenable to intervention.

A 2018 systematic review by Wopken et al. identified pre-treatment weight loss, advanced T- and N-category, concurrent chemotherapy, prophylactic gastrostomy policy, and dose-volume histogram (DVH) parameters of the larynx, pharyngeal constrictors and contralateral parotid gland as the most consistent predictors of tube dependence at six months (10). The phase III DARS trial subsequently established Level I evidence that dysphagia-optimised intensity-modulated radiotherapy (DO-IMRT), with mean-dose constraints on the constrictors and supraglottic larynx, improves patient-reported swallowing function relative to standard IMRT (11). Behavioural factors are equally consequential: a meta-analysis of more than 2,300 patients demonstrated that post-diagnosis smoking cessation confers a significant overall-survival benefit (summary HR 0.80) (12), and continued smoking during radiotherapy is independently associated with reduced locoregional control and elevated late toxicity (13, 14).

Despite this expanding evidence base, most published cohorts are heterogeneous in radiation technique, chemotherapy regimen, gastrostomy policy and follow-up duration, limiting external validity. Few studies have integrated clinical and dosimetric predictors with a survival endpoint in a single, uniformly treated population. Selected contemporary studies are summarised in **Table 1**.

**Table 1:** Selected key studies on predictors of feeding tube dependence and dosimetric sparing of swallowing structures in head and neck cancer.

Study (year)/design	Population	Question/exposure	Key findings	Clinical take-home
Nutting et al., Lancet Oncol 2023 (DARS, phase III RCT) (11)	112 patients with pharyngeal SCC randomised to DO-IMRT vs standard IMRT.	Effect of constrictor and supraglottic-larynx sparing on swallowing function.	Mean dose ≤ 50 Gy to constrictors outside the high-dose PTV improved MDADI composite score at 12 months without compromising disease control.	Provides Level I evidence for routine constrictor sparing — should anchor every modern HNC plan.
Wopken et al., Radiother Oncol 2018 (systematic review) (10)	Pooled analysis spanning > 4,000 HNC patients across 22 cohorts.	Clinical and dosimetric predictors of tube dependence at ≥ 6 months.	Consistent predictors: pre-treatment weight loss, advanced T/N, concurrent chemo, prophylactic gastrostomy policy, and DVH parameters of larynx, constrictors and contralateral parotid.	Endorses an integrated clinical–dosimetric prediction framework rather than single-variable triggers.
Caini et al., Br J Cancer 2022 (meta-analysis) (12)	16 studies, > 2,300 HNC patients.	Effect of post-diagnosis smoking cessation on overall survival.	Quitters at/around diagnosis had significantly better OS than continued smokers (summary HR 0.80; 95% CI 0.70–0.91).	Cessation programmes should be embedded in standard HNC oncologic care pathways.
Smith et al., Radiother Oncol 2019 (systematic review & meta-analysis) (13)	Pooled HNC patients receiving radiotherapy with documented smoking status.	Effect of continued smoking during RT on OS, locoregional control and toxicity.	Continued smoking associated with reduced OS, lower locoregional control and higher late toxicities.	Clinicians should counsel cessation at every visit; consider biomarker-confirmed abstinence.
Setton et al., Cancer 2015 (multi-institution pooled, IMRT era) (21)	421 HNC patients (66% HPV-positive oropharyngeal) treated with definitive IMRT/CRT.	Trends in feeding tube use by HPV status in modern IMRT practice.	Feeding tube use in HPV-positive OPC declined from 80% (2007) to 24% (2014); only 10% remained tube-dependent beyond 90 days.	Reactive (vs prophylactic) gastrostomy is reasonable in low-risk HPV-positive OPC managed with IMRT.
Strom et al., JAMA Otolaryngol 2013 (single-centre cohort) (8)	297 oropharyngeal SCC patients treated with IMRT + chemo without upfront PEG.	Risk factors for reactive PEG placement during/within 3 months of CRT.	11.7% required reactive PEG; independent risks: BMI < 25, T3–T4, accelerated fractionation, cisplatin ≥ 200 mg/m².	Routine prophylactic PEG is unnecessary in low-risk OPSCC; reserve for high-risk constellations.
Friedes et al., Cureus 2020 (single-centre cohort) (9)	61 unresected stage III–IVB HNSCC patients treated with definitive RT/CST.	Association of late PEG dependency with overall survival.	Late PEG dependence was independently associated with worse OS (log-rank p = 0.022).	Prolonged dependence is a clinically and prognostically meaningful endpoint, not merely a supportive-care metric.

CRT, chemoradiotherapy; CST, concurrent systemic therapy; DARS, Dysphagia/Aspiration at Risk Structures; DO-IMRT, dysphagia-optimised intensity-modulated radiotherapy; DVH, dose–volume histogram; HNC, head and neck cancer; HNSCC, head and neck squamous cell carcinoma; HPV, human papillomavirus; IMRT, intensity-modulated radiotherapy; MDADI, MD Anderson Dysphagia Inventory; NTCP, normal tissue complication probability; OAR, organ at risk; OPC, oropharyngeal cancer; OS, overall survival; PEG, percutaneous endoscopic gastrostomy; PTV, planning target volume; RT, radiotherapy; SCC, squamous cell carcinoma; VA, Veterans Affairs.

Studies were selected to represent contemporary, methodologically rigorous evidence on (i) clinical predictors of long-term tube dependence, (ii) dosimetric thresholds for swallowing-related structures, and (iii) the impact of smoking and HPV status on outcomes.

We report the largest single-institution analysis to date of LAHNC patients managed with a uniform protocol of curative-intent chemoradiotherapy to 70 Gy and prophylactic gastrostomy. Our objectives were threefold: (i) to quantify the prognostic relevance of prolonged feeding tube dependence on overall survival; (ii) to identify clinical and dosimetric predictors of prolonged dependence; and (iii) to delineate modifiable factors — particularly tobacco exposure and dose to critical swallowing structures — that can be acted upon in routine practice.

## 2 Materials and methods

### 2.1 Study design and patients

This was a single-centre, retrospective cohort study of consecutive adult patients with histologically confirmed LAHNC treated at the McGill University Health Centre between January 2015 and December 2022. Eligibility criteria were: (i) curative-intent radiotherapy to 70 Gy with concurrent systemic therapy; (ii) prophylactic PEG or RIG insertion either before treatment or within the first week of radiotherapy; and (iii) availability of complete clinical, dosimetric and follow-up data. Patients receiving palliative radiotherapy, those without prophylactic tube placement, and those with synchronous distant metastases were excluded. The Institutional Ethics Committee approved the study and granted a waiver of informed consent given the retrospective design.

### 2.2 Supportive care and tube removal

All patients underwent routine nutritional assessment by the departmental oncology dietitian. Gastrostomy removal was not governed by a written institutional protocol but followed a consistent dietitian-led practice: once a patient was eating and drinking exclusively by mouth, removal was requested after body weight had remained stable for 4 weeks. Referral to speech-language pathology was at the treating clinician’s discretion.

### 2.3 Radiotherapy and systemic therapy

All patients were planned with intensity-modulated radiotherapy (IMRT) or volumetric-modulated arc therapy (VMAT) to a total dose of 70 Gy in 33–35 fractions, delivered concurrently with platinum-based chemotherapy or, for selected cases, cetuximab; as sole definitive treatment, with no prior surgery and no neoadjuvant or adjuvant surgical component. Target volumes followed institutional guidelines, with high-, intermediate- and low-risk planning target volumes (PTVs) prescribed to 70 Gy, 63 Gy and 56 Gy respectively. Organs at risk (OAR) — including the oral cavity, lips, mandible, parotid glands, larynx and superior, middle and inferior pharyngeal constrictor muscles — were contoured according to standard atlases, and mean and maximum doses were extracted from the planning system.

### 2.4 Variables and definitions

Demographic and disease variables included age, sex, primary tumour site, TNM stage (AJCC 7th edition), HPV status (p16 immunohistochemistry for oropharyngeal cases), Karnofsky Performance Status (KPS) at treatment initiation, body mass index (BMI), and smoking history. Smoking exposure was categorised as never-smoker (lifetime exposure < 1 pack-year), past smoker (cessation prior to therapy) or current smoker (continued smoking during treatment). Toxicity was monitored weekly during radiotherapy and included assessment of mucositis, dermatitis, dysphagia, KPS and weight.

The primary endpoint was duration of feeding tube dependence, calculated from the first day of radiotherapy until documented tube removal. Prolonged dependence was defined *a priori* as duration exceeding the cohort median of 3.2 months. Overall survival (OS) was calculated from the start of treatment until death from any cause or the censor date (1 February 2024).

As a sensitivity analysis anchored to the conventional clinical endpoint, persistent tube dependence at 6 months was modelled with multivariable logistic regression in patients with a gastrostomy who survived beyond 6 months, using the same covariates as the primary model.

### 2.5 Statistical analysis

Given that feeding-tube duration is a time-to-event variable subject to informative censoring, the primary endpoint was redefined as time from the start of radiotherapy to documented feeding-tube removal, analysed using Kaplan-Meier methodology and multivariable Cox proportional-hazards regression, rather than logistic regression on a dichotomised outcome. Patients who died before tube removal were treated as a competing risk; cause-specific Cox regression (censoring at death) was performed as the primary model, and a Fine-Gray subdistribution-hazard model was fitted as a sensitivity analysis to confirm that results were not materially affected by this competing event. Dosimetric and functional covariates (constrictor muscle, oral cavity, lip and mandible mean doses; baseline KPS) were modelled as continuous variables rather than dichotomised at the cohort mean. For the overall-survival analysis, because tube-dependence status is itself a time-dependent exposure originating from the same time zero as the survival outcome, the original baseline-group comparison is susceptible to guarantee-time (immortal-time) bias. This was addressed using a landmark analysis: patients surviving to 3, 6 and 12 months post-treatment were classified according to their tube status at each landmark, and overall survival from that landmark onward was compared by group using the log-rank test and Cox regression. Landmark analysis was restricted to patients who received a PEG/RIG tube (n=204; 213 minus the 9 with inconsistent extraction dates), since dependence cannot be defined in patients who never had a tube. At each landmark, the association was further estimated in a multivariable Cox model adjusted for baseline KPS, T classification and N classification (both ordinal), any smoking history and age at treatment start, and covariate-adjusted survival curves were derived from this model (**Figure 1**).

**Figure 1:**
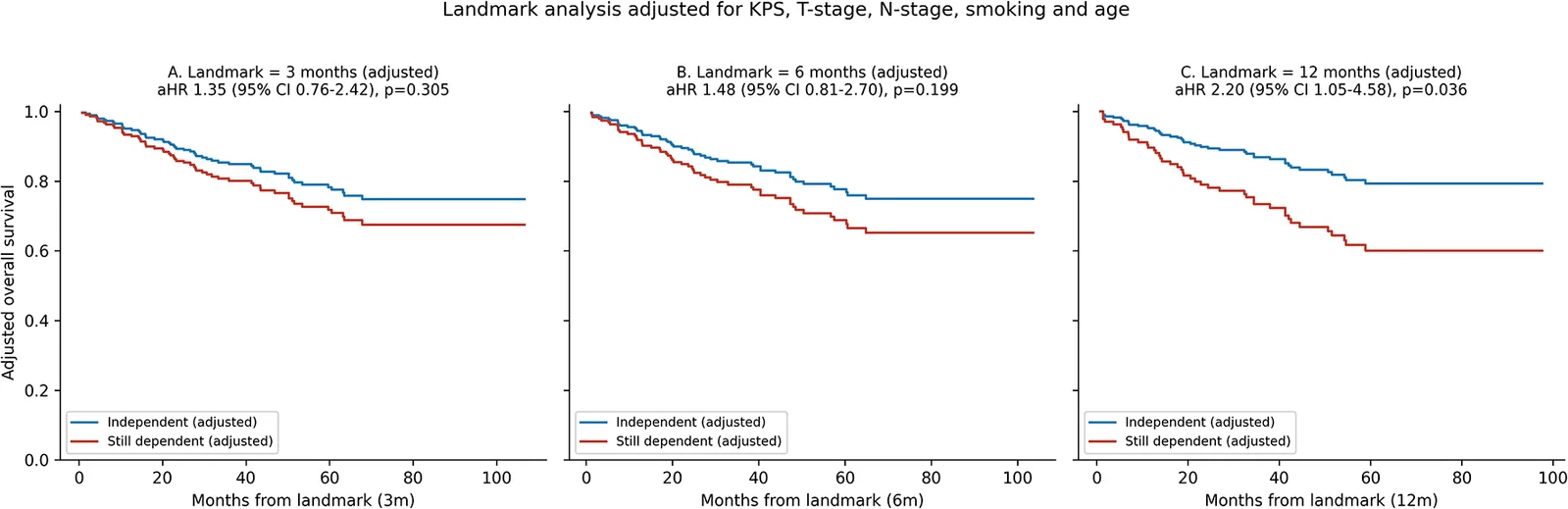
Covariate-adjusted overall survival according to feeding-tube status at the 3-, 6- and 12-month landmarks in patients treated with a prophylactic gastrostomy (n = 204). Patients alive at each landmark were classified as tube-independent or still tube-dependent based on tube status known at that time point, and survival was measured from the landmark onward. Curves were derived from a multivariable Cox model including tube status, baseline KPS, T classification, N classification, smoking history and age at treatment start. **(A)** 3-month landmark: 200 patients at risk, 106 still dependent; aHR 1.35 (95% CI 0.76–2.42), p = 0.305. **(B)** 6-month landmark: 194 at risk, 51 still dependent; aHR 1.48 (95% CI 0.81–2.70), p = 0.199. **(C)** 12-month landmark: 186 at risk, 27 still dependent; aHR 2.20 (95% CI 1.05–4.58), p = 0.036. Unadjusted estimates are reported in **Table 4**, and the corresponding unadjusted Kaplan–Meier curves in **Supplementary Figure S1**. aHR, adjusted hazard ratio; CI, confidence interval; KPS, Karnofsky performance status.

## 3 Results

### 3.1 Cohort characteristics

A total of 213 patients met the inclusion criteria, nine were excluded from the time-to-independence analysis because their recorded tube-extraction date fell after their date of death — a logical inconsistency that could not be resolved against the source medical record (see Methods). The remaining 204 patients constitute the analytic cohort for the survival analyses below. Median age was 64 years and 77% were male. The oropharynx was the predominant primary site (65%), followed by the larynx (16%), nasopharynx (7.0%), oral cavity (5%) and other subsites (7.0%). T3–T4 disease was present in 54%, and 80% had nodal involvement. Of the 132 oropharyngeal patients, 131 (99%) had documented HPV status, of whom 82% were HPV-positive. Baseline characteristics of this cohort are presented in **Table 2**.

**Table 2:** Baseline characteristics of the analytic cohort (n=204).

Characteristic	Category	n (%)
Sex	Male	157 (77)
	Female	47 (23)
Age (years), median 64	> 64	102 (50)
	<= 64	102 (50)
Karnofsky Performance Status	>= 90	125 (61)
	< 90	79 (39)
Smoking history	Ever smoker	153 (75)
	Never smoker	50 (25)
Smoking activity (ever-smokers, n=99)	Past smoker (quit before RT)	51 (52)
	Current smoker (during RT)	48 (48)
Primary tumour site	Oropharynx	132 (65)
	Larynx	32 (16)
	Hypopharynx	6 (3)
	Sinu-Nasal	3 (1)
	Nasopharynx	15 (7)
	Oral Cavity	11 (5)
	Unknown Primary	5 (2)
T classification	T0/Tis	13 (6)
	T1	29 (14)
	T2	52 (25)
	T3	53 (26)
	T4	57 (28)
N classification	N0	40 (20)
	N1	23 (11)
	N2	128 (63)
	N3	13 (6)
HPV status (oropharyngeal subset, n=131 tested of 132)	Positive	108 (82)
	Negative	23 (18)

Values are n (%). Cohort reflects the 204 patients analysed for time to feeding-tube independence (213 patients with a PEG/RIG tube, minus 9 excluded due to a logically inconsistent extraction date - see Methods). This table is descriptive only; the prior version stratified by the >3.2-month dichotomised dependence threshold has been moved to **Supplementary Table S1** for transparency, since the primary outcome is now modelled continuously via Cox regression rather than as a binary threshold. Smoking-activity sub-classification (past vs. current) was available for a subset of ever-smokers only, as in the original cohort. HPV status assessed by p16 immunohistochemistry on the oropharyngeal subset only.

### 3.2 Time to feeding-tube independence

Median time to feeding-tube independence was 3.2 months (**Figure 2A**). Of the 204 patients, 199 achieved documented tube removal; five patients died before ever achieving independence and were treated as a competing risk rather than as censored observations, since death precludes the possibility of the primary event occurring (Aalen–Johansen cumulative incidence, **Figure 2B**).

**Figure 2:**
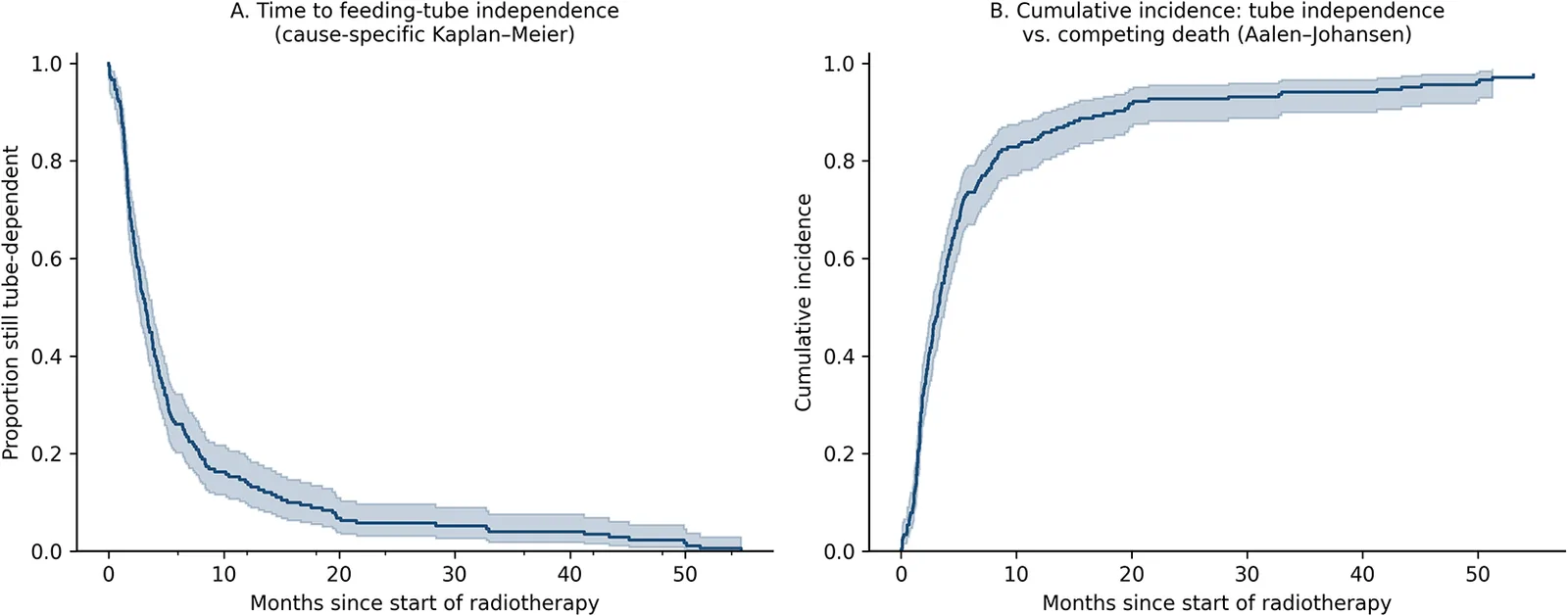
Time to feeding-tube independence (n=204). **(A)** Cause-specific Kaplan–Meier estimate of the proportion of patients remaining feeding-tube dependent over time since the start of radiotherapy, censoring at death (median time to independence 3.2 months; shaded band, 95% CI). **(B)** Aalen–Johansen cumulative incidence of feeding-tube independence treating death before independence as a competing risk; five patients died before ever achieving independence and are censored in **(A)** but contribute to the competing event in **(B)**. The two panels are shown together to illustrate that the competing-risk treatment of death does not materially change the independence curve, consistent with the low competing-event rate (5/204).

### 3.3 Multivariable Cox model for time to feeding-tube independence

Because dependence duration is a time-to-event variable subject to informative censoring, predictors were evaluated using multivariable Cox proportional-hazards regression on the continuous duration, rather than logistic regression on a dichotomised threshold (**Table 3**; **Figure 3**). Any history of smoking remained independently associated with slower attainment of tube independence (HR 0.60, 95% CI 0.42–0.84; p = 0.004), as did higher baseline KPS, which was associated with faster independence (HR 1.02 per point, 95% CI 1.01–1.04; p = 0.006). Mean dose to the middle pharyngeal constrictor remained independently associated with slower independence when modelled continuously (HR 0.98 per Gy, 95% CI 0.96–1.00; p = 0.048); mean oral cavity dose showed a similar but non-significant trend (HR 0.98 per Gy, 95% CI 0.95–1.00; p = 0.067). Mean doses to the superior constrictor, lips and mandible were not significant. A Fine–Gray subdistribution-hazard model, fitted as a sensitivity analysis to account for death as a competing risk, produced consistent effect sizes and significance for smoking (p = 0.010), middle constrictor dose (p = 0.011) and oral cavity dose (p = 0.019), supporting the robustness of these associations (**Supplementary Table S2**). Univariable logistic-regression estimates from the original dichotomised analysis are retained in **Supplementary Table S1** for comparison.

**Table 3:** Multivariable Cox proportional-hazards model for time to feeding-tube independence.

Variable	HR	95% CI	p-value
**Baseline KPS (continuous)**	**1.02**	**1.01-1.04**	**0.006**
**Any smoking history (1 = yes)**	**0.60**	**0.42-0.84**	**0.004**
Superior constrictor mean dose (Gy)	0.99	0.96-1.03	0.581
**Middle constrictor mean dose (Gy)**	**0.98**	**0.96-1.00**	**0.048**
Oral cavity mean dose (Gy)	0.98	0.95-1.00	0.067
Lips mean dose (Gy)	0.98	0.95-1.02	0.299
Mandible mean dose (Gy)	1.01	0.98-1.04	0.527

HR > 1 indicates faster tube independence (shorter dependence). Model fitted on n=197 patients with complete predictor data (cause-specific hazard; death before independence censored). Baseline KPS and dosimetric variables are modelled continuously (HR per unit/per Gy). Bold rows: p<0.05.

**Figure 3:**
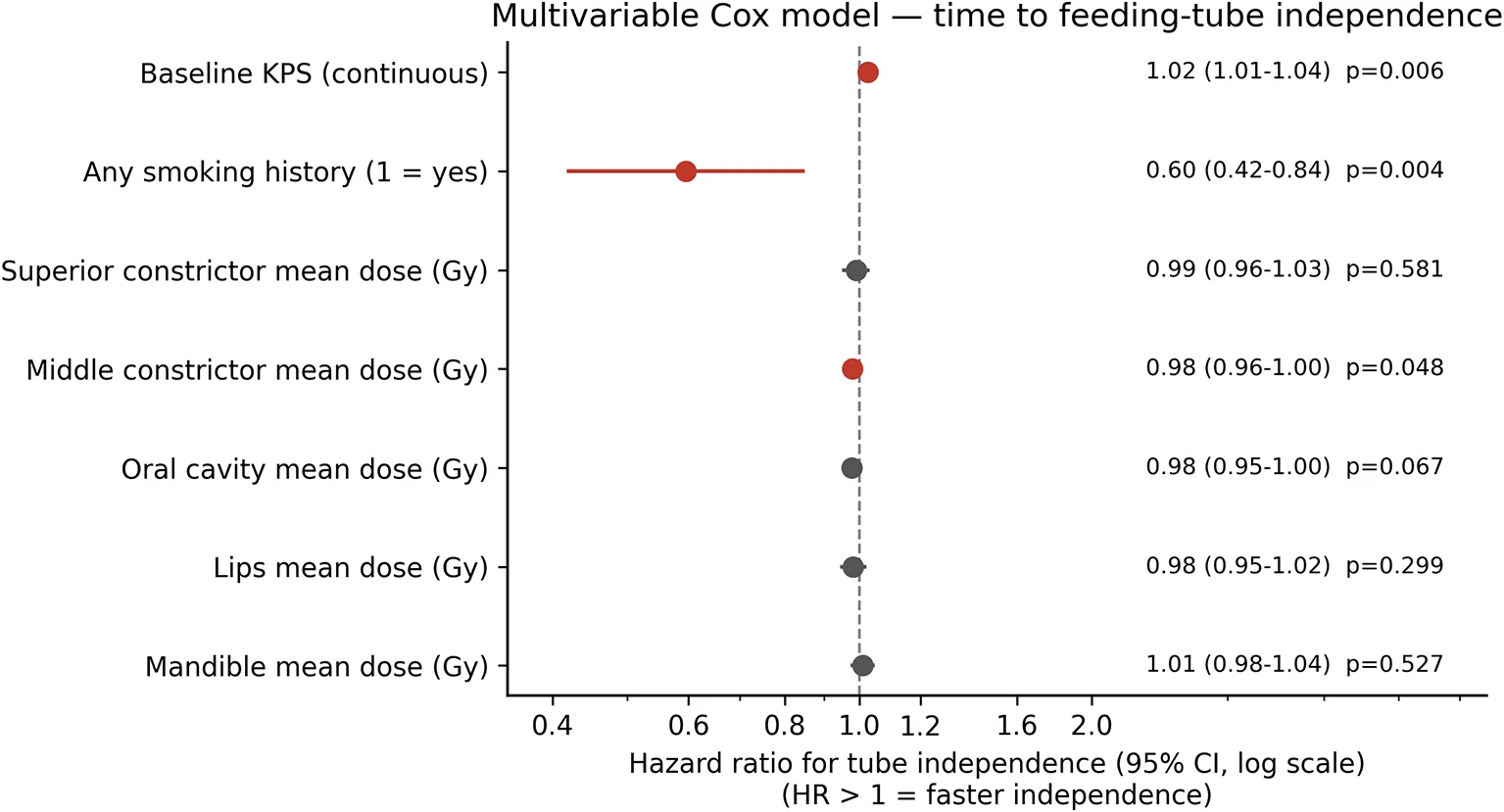
Forest plot of the multivariable Cox proportional-hazards model for time to feeding-tube independence (**Table 3**; n=197 with complete predictor data). Points show hazard ratios (HR) with 95% confidence intervals on a log-scaled axis; HR > 1 indicates faster attainment of independence (shorter dependence). Baseline KPS and all dosimetric variables are modelled continuously (HR per point/per Gy) rather than dichotomised. Red markers denote covariates significant at p<0.05: any smoking history was associated with slower independence, while higher baseline KPS and lower mean dose to the middle pharyngeal constrictor were associated with faster independence; oral cavity dose showed a non-significant trend in the same direction.

To align with the conventional clinical endpoint, we also modelled persistent dependence at 6 months. Of the 188 patients with a gastrostomy who survived beyond 6 months and had complete predictor data, 49 (26.1%) remained tube-dependent. On multivariable logistic regression, baseline KPS was the only significant predictor, with each additional point associated with lower odds of persistent dependence (OR 0.96, 95% CI 0.92–1.00; p = 0.037). Smoking history (OR 1.64, 95% CI 0.69–3.86; p = 0.260) and middle constrictor dose (OR 1.04 per Gy, 95% CI 0.99–1.10; p = 0.126) pointed in the same direction as in the time-to-event model but did not reach significance. Oral cavity dose showed a borderline association (OR 1.05 per Gy, 95% CI 1.00–1.10; p = 0.074) (**Supplementary Table S3**).

### 3.4 Overall survival: landmark analysis

Because feeding-tube status and overall survival are both time-dependent variables measured from the same time origin, the original baseline-group comparison of OS is susceptible to guarantee-time (immortal-time) bias: an individual can only be classified as “prolonged-dependent” by surviving long enough to remain tube-dependent past the threshold. This was addressed using a landmark analysis, in which tube status was fixed at 3, 6 and 12 months post-treatment using only information available up to that point, and survival was compared from each landmark forward. At the 3-, 6- and 12-month landmarks, 200, 194 and 186 patients were at risk, of whom 106, 51 and 27 remained tube-dependent. Patients still tube-dependent at 6 months had worse subsequent survival than those who had already achieved independence (unadjusted HR 1.78, 95% CI 1.01–3.13; p = 0.048), and this association strengthened at the 12-month landmark (unadjusted HR 2.53, 95% CI 1.30–4.91; p = 0.006); the 3-month landmark showed a consistent trend that did not reach significance in the unadjusted model (HR 1.71, 95% CI 0.98–3.00; p = 0.059) (**Supplementary Figure S1**). Because tube dependence could plausibly be a surrogate for tumour burden, performance status or smoking rather than an independent driver of mortality, we repeated each landmark comparison in a multivariable Cox model adjusting for baseline KPS, T classification, N classification, smoking history and age (**Table 4**; **Figure 1**). After adjustment, the association at the 3- and 6-month landmarks was attenuated and no longer significant (adjusted HR 1.35, 95% CI 0.76–2.42; p = 0.305, and adjusted HR 1.48, 95% CI 0.81–2.70; p = 0.199, respectively). Patients still dependent at 12 months, however, retained roughly twice the hazard of death of those who had become independent (adjusted HR 2.20, 95% CI 1.05–4.58; p = 0.036). This 12-month estimate rests on 27 still-dependent patients, with an events-per-variable ratio of 7.3, and its confidence interval is correspondingly wide.

**Table 4:** Landmark analysis of overall survival according to feeding-tube status at 3, 6 and 12 months after treatment start in patients who received a prophylactic gastrostomy (n = 204).

Landmark (months)	N at risk/still dependent	Unadjusted analysis			Adjusted analysis (KPS, T, N, smoking, age)		
		HR	95% CI	p-value	aHR	95% CI	p-value
3	200/106	1.71	0.98-3.00	0.059	1.35	0.76-2.42	0.305
6	194/51	1.78	1.01-3.13	**0.048**	1.48	0.81-2.70	0.199
12	186/27	2.53	1.30-4.91	**0.006**	2.20	1.05-4.58	**0.036**

Hazard ratios compare still-dependent with tube-independent patients (reference). “N at risk” is the number of patients alive beyond the landmark with complete covariate data. “Still dependent” is the number who had not achieved tube independence by the landmark, including 5 patients who later died with the tube in place. The adjusted model includes baseline KPS, T classification and N classification (both ordinal), any smoking history, and age at treatment start. Nine patients whose recorded extraction date followed their date of death were excluded (see Methods). Events-per-variable at the 12-month landmark was 7.3, so this estimate should be interpreted with caution. Bold values indicate p < 0.05. HR, hazard ratio; aHR, adjusted hazard ratio; CI, confidence interval; KPS, Karnofsky performance status.

## 4 Discussion

In this homogeneous cohort of 204 patients with LAHNC treated to 70 Gy with concurrent systemic therapy and prophylactic gastrostomy, feeding-tube dependence persisting to 12 months was associated with worse subsequent survival, independent of T classification, N classification, baseline performance status, smoking history and age. At the 3- and 6-month landmarks, the unadjusted survival difference was largely accounted for by these baseline factors. Time to independence itself was independently predicted by smoking history, baseline KPS and radiation dose to the middle pharyngeal constrictor, with a similar trend for oral cavity dose. To our knowledge, this is the largest single-institution analysis to integrate clinical and dosimetric predictors of tube-independence with a confounder-adjusted, bias-corrected survival endpoint in a uniformly managed prophylactic-gastrostomy cohort, and it identifies two actionable, modifiable levers for routine practice.

### 4.1 Dependence at one year carries prognostic weight

The association between prolonged dependence and inferior OS is consistent with — and extends — the observation by Friedes et al. in a smaller cohort, where late PEG dependency was independently linked to worse OS (9). In contrast, large database studies have shown that the *timing* of tube placement (prophylactic vs reactive) does not, by itself, modify OS once allocation bias is controlled (7). Taken together, the contemporary evidence suggests that the survival signal is driven not by gastrostomy *placement* but by the underlying biology and toxicity that *perpetuates* dependence — namely, severe and persistent dysphagia, aspiration risk, sarcopenia, and continued tobacco exposure. The attenuation we saw at 3 and 6 months fits this reading. Early dependence is common, and at that stage it largely mirrors how unwell the patient was at baseline; once stage, KPS, smoking and age were accounted for, it no longer separated survival. Dependence that persists to one year is different. It identifies a smaller group whose excess mortality is not explained by those factors. Observational data cannot tell us whether shortening dependence would itself improve survival, but reducing its duration remains a clear quality-of-life goal, and patients still tube-dependent at 12 months may warrant closer surveillance.

### 4.2 Smoking: the most modifiable patient-level driver

Smoking history was the strongest independent clinical predictor in our analysis: ever-smokers reached tube independence at a roughly 40% lower rate than never-smokers (HR 0.60). Importantly, this risk was apparent even in patients who had stopped smoking before therapy compared with never-smokers, mirroring meta-analytic data showing reduced OS, lower locoregional control and higher late toxicity among smokers (13). Two complementary mechanisms are proposed. First, chronic smoking induces tumour hypoxia and microvascular compromise, attenuating radiation efficacy and impairing mucosal healing. Second, smokers exhibit poorer baseline nutritional and pulmonary reserves, predisposing them to slower swallowing recovery (12–14). The clinical implication is straightforward: structured smoking cessation programmes — ideally initiated within six months of diagnosis, where evidence-based intervention has demonstrated maximal survival benefit (14) — should be considered a mandatory component of curative head and neck oncology, on a par with nutritional and dental optimisation. The 6-month analysis tells a partly different story from the time-to-event model. Baseline performance status was the only factor that clearly separated patients still dependent at 6 months, while smoking and constrictor dose lost significance. We would not read this as evidence that smoking and dose matter only early in recovery. A binary 6-month endpoint discards the timing of recovery for every patient, and with 49 events the model has limited power. The odds ratios for smoking, middle constrictor dose and oral cavity dose all point the same way as in the primary analysis. A more plausible reading is that baseline condition largely determines who is still dependent at a fixed time point, whereas smoking and swallowing-structure dose shape how quickly patients get there. This is also why we kept the continuous time-to-event model as the primary analysis.

### 4.3 Dose to the middle constrictor and oral cavity: the dominant dosimetric levers

The independent association of middle pharyngeal constrictor dose, and to a lesser extent oral cavity dose, with time to independence is biologically and dosimetrically coherent. The phase III DARS trial demonstrated that a mean-dose constraint of ≤ 50 Gy on the pharyngeal constrictors lying outside the high-dose target volume translates into a clinically meaningful improvement in patient-reported swallowing function at 12 months (10). Subsequent dose–volume modelling has highlighted the middle constrictor in particular: Huynh et al. identified mean dose to the middle pharyngeal constrictor as an independent predictor of long-term dysphagia in HNC survivors (15), while Schwartz, Eisbruch and others have established the broader principle that DVH parameters of the constrictors and supraglottic larynx forecast late aspiration and feeding tube dependence (16, 17). We did not derive a dose threshold from our own data. The continuous association we observed, roughly a 2% lower rate of independence per additional Gy, cannot be mapped directly onto the DARS 50 Gy target, particularly when the primary target is itself oropharyngeal, but it is consistent with the principle that *any incremental sparing matters*.

The oral cavity signal was weaker: it did not reach significance in the primary cause-specific model (p = 0.067) but was significant in the Fine–Gray sensitivity analysis (**Supplementary Table S2**). Oral cavity exposure compromises mastication, salivary mixing and bolus transit, and contributes to xerostomia-associated dysphagia. Recent narrative and dosimetric reviews have called for routine integration of oral cavity contouring and dose constraints into modern DO-IMRT and proton-therapy workflows (18), and our findings provide direct outcomes-level support for that recommendation. In contrast to several earlier series, the inferior pharyngeal constrictor dose did not reach independent significance in our cohort (19, 20), likely because most patients had primary disease at or above the supraglottic plane, where superior and middle constrictor dose dominates the toxicity signal.

### 4.4 HPV status, performance status and the favourable subgroup

HPV-positive oropharyngeal carcinoma was associated with shorter dependence in our cohort, in line with the observation by Setton et al. of a dramatic decline in feeding tube use in HPV-positive disease in the IMRT era (21). Although Vangelov et al. previously suggested higher tube *requirement* in HPV-positive patients (22), this likely reflects differing institutional thresholds for tube placement; our prophylactic-tube design controls for that selection bias and instead isolates *recovery* trajectory, where the younger age, lower comorbidity and better tumour responsiveness of HPV-positive disease appear to confer faster functional recovery. Higher KPS at baseline was likewise protective, consistent with broad evidence that pre-treatment functional reserve is among the most reliable predictors of post-treatment recovery (10).

### 4.5 Towards an integrated risk-adapted strategy

Several validated prediction models — most notably those by Wopken et al. and Brown et al. — combine pre-treatment weight loss, T- and N-stage, bilateral neck irradiation, accelerated fractionation and chemoradiation use to estimate tube-feeding dependence at 6 months, with AUCs of 0.82–0.86 (10, 23). Our data complement these models by incorporating *intra-treatment* dosimetric information that becomes available once planning is finalised, allowing iterative re-optimisation if dose to the middle constrictor or oral cavity is higher than necessary. Embedding such a two-step risk stratification (pre-treatment clinical model + on-plan dosimetric review) into the standard workflow could meaningfully reduce the burden of long-term dependence.

### 4.6 Strengths and limitations

Strengths include the homogeneous treatment paradigm (uniform 70 Gy chemoradiation with prophylactic gastrostomy), the standardised contouring and dosimetry, and the integration of clinical, dosimetric and survival endpoints in a single analytical framework corrected for immortal-time bias and adjusted for known confounders. Several limitations warrant explicit acknowledgement. First, and most importantly, no data were available on speech-language pathology intervention, prophylactic swallowing exercises, or rehabilitation compliance during and after treatment and SLP referral was at clinician discretion rather than protocolised. Time to feeding-tube removal is directly influenced by the intensity and timing of swallowing therapy, and institutional referral practice for this was not captured in this dataset; it is plausible that some of the association we attribute to dose or smoking is partly mediated by differential access to or engagement with swallowing rehabilitation, and this should be regarded as the principal unmeasured confounder in this analysis. Second, disease control outcomes (local, regional and distant recurrence) were not captured. The dataset was assembled from radiotherapy planning and on-treatment records, and post-treatment surveillance was not protocolised across the study period. Retrospective ascertainment of recurrence would therefore depend on non-uniform imaging schedules and follow-up locations, and would risk introducing its own misclassification. Recurrent or persistent disease can prolong tube dependence and independently shorten survival, so we cannot exclude that it contributes to the 12-month association. The adjusted 12-month landmark estimate also rests on only 27 still-dependent patients, with an events-per-variable ratio of 7.3, below the conventional threshold of about 10; its direction agrees with the unadjusted analysis, but its magnitude should be regarded as imprecise.

Third, the cohort was restricted to patients completing definitive chemoradiotherapy or radiotherapy with cetuximab to 70 Gy as sole curative-intent treatment, with prophylactic (not reactive) gastrostomy; findings may not generalise to institutions that favour reactive PEG placement, to patients treated with surgery followed by adjuvant or neoadjuvant therapy, or to lower-dose palliative-intent regimens. Other unmeasured confounders inherent to retrospective single-centre design include alcohol intake, sarcopenia, and granular smoking exposure (pack-years and cessation interval, rather than the ever/never/current categorisation available in this dataset), along with a moderate sample size for some subgroups (notably HPV-negative oropharyngeal and oral cavity cancers) and the absence of prospectively collected patient-reported swallowing outcomes such as the MD Anderson Dysphagia Inventory. External multicentre validation, ideally embedded within prospective DO-IMRT trials that also capture swallowing-therapy intensity and disease control endpoints, is warranted. The 6-month logistic model had approximately 7 events per predictor and is best regarded as a sensitivity analysis. Its non-significant estimates for smoking and dose more likely reflect limited power than the absence of an effect.

### 4.7 Conclusion

Feeding-tube dependence persisting to 12 months in patients with LAHNC undergoing prophylactic gastrostomy and curative chemoradiotherapy is associated with inferior overall survival, independent of tumour stage, nodal stage, baseline performance status, smoking history and age; at 3 and 6 months, the association is largely explained by these baseline factors. Time to independence itself is predicted by two modifiable factors: smoking history, and radiation dose to the middle pharyngeal constrictor (with a similar trend for oral cavity dose), modelled continuously rather than at an arbitrary cut point. Embedding pre-treatment smoking-cessation programmes and dysphagia-optimised planning that explicitly minimises dose to the middle constrictor and oral cavity into routine multidisciplinary care has the potential to reduce long-term tube dependence in this population. Prospective, externally validated dose thresholds and multicentre confirmation, ideally within trials incorporating patient-reported swallowing endpoints, are now warranted.

## Data Availability

The raw data supporting the conclusions of this article will be made available by the authors, without undue reservation.
