# Supplementary material for: Predictors of time to feeding-tube independence in locally advanced head and neck cancer
Source: Front Oncol. 2026 Oct 20;16:1956034. doi: 10.3389/fonc.2026.1956034 (PMC13727488; doi:10.3389/fonc.2026.1956034)
Supplement: Supplementary file 1 [file DataSheet1.docx]

**Supplementary material:**

**Table S1. Baseline Characteristics by Feeding Tube Duration**

| **Characteristic** | **≤ 3.3 months (n = 106; 49.8%)** | **> 3.3 months (n = 107; 50.2%)** | **p value** |
| --- | --- | --- | --- |
| ***Sex*** |  |  |  |
| Male | 88 (83) | 77 (72) | 0.053 |
| Female | 18 (17) | 30 (28) |  |
| ***Age (years)*** |  |  |  |
| > 63 | 52 (49) | 60 (56) | 0.30 |
| ≤ 63 | 54 (51) | 47 (44) |  |
| ***Karnofsky Performance Status*** |  |  |  |
| > 90 | 73 (69) | 52 (49) | 0.003 |
| ≤ 90 | 33 (31) | 55 (51) |  |
| ***Smoking history*** |  |  |  |
| Ever smoker | 72 (68) | 89 (83) | 0.011 |
| Never smoker | 34 (32) | 18 (17) |  |
| ***Smoking activity (ever-smokers)*** |  |  |  |
| Past smoker (quit before RT) | 78 (74) | 81 (76) | 0.54 |
| Current smoker (during RT) | 28 (26) | 26 (24) |  |
| ***Primary tumour site*** |  |  |  |
| Oropharynx | 69 (66) | 67 (63) | 0.18 |
| Larynx | 21 (20) | 12 (11) |  |
| Nasopharynx | 8 (8) | 9 (8) |  |
| Oral cavity | 3 (2) | 9 (8) |  |
| Other sites | 5 (4) | 10 (10) |  |
| ***T classification (AJCC 7th ed.)*** |  |  |  |
| T1 | 17 (16) | 12 (11) | 0.51 |
| T2 | 28 (26) | 25 (23) |  |
| T3 | 33 (31) | 21 (20) |  |
| T4 | 23 (22) | 41 (38) |  |
| Tx / Tis | 5 (5) | 8 (8) |  |
| ***N classification (AJCC 7th ed.)*** |  |  |  |
| N0 | 24 (23) | 18 (17) | 0.53 |
| N1 | 11 (10) | 12 (11) |  |
| N2 | 63 (59) | 72 (67) |  |
| N3 | 8 (8) | 5 (5) |  |
| ***HPV status (oropharyngeal subset)*** |  |  |  |
| Positive | 65 (88) | 55 (74) | 0.039 |
| Negative | 9 (12) | 19 (26) |  |

Values are n (%). p values are derived from Pearson’s χ² test. Bold p values denote statistical significance (p < 0.05). HPV status was assessed by p16 immunohistochemistry on the oropharyngeal subset only.

Abbreviations: AJCC, American Joint Committee on Cancer; HPV, human papillomavirus; KPS, Karnofsky Performance Status; RT, radiotherapy.

| **Table S2. Fine-Gray-equivalent subdistribution-hazard model (competing-risk sensitivity analysis)** | | | |
| --- | --- | --- | --- |
|  |  |  |  |
| **Variable** | **HR** | **95% CI** | **p-value** |
| **Baseline KPS (continuous)** | **1.03** | **1.01-1.04** | **0.006** |
| **Any smoking history (1 = yes)** | **0.64** | **0.46-0.90** | **0.010** |
| Superior constrictor mean dose (Gy) | 1.00 | 0.97-1.04 | 0.860 |
| **Middle constrictor mean dose (Gy)** | **0.98** | **0.96-0.99** | **0.011** |
| **Oral cavity mean dose (Gy)** | **0.97** | **0.95-1.00** | **0.019** |
| Lips mean dose (Gy) | 1.00 | 0.96-1.04 | 0.971 |
| Mandible mean dose (Gy) | 1.00 | 0.97-1.03 | 0.786 |
|  |  |  |  |
| *Sensitivity analysis treating death with the feeding tube still in place as a competing risk (weighted Cox implementation of the Fine & Gray, 1999 subdistribution hazard). n=197. HR > 1 indicates faster tube independence. Results are directionally and statistically consistent with the primary cause-specific model (Table 3), supporting robustness of the findings to the competing-risk of death.* | | | |

**Table S3. Multivariable logistic regression predicting persistent tube dependence at the 6-month landmark**

| **Variable** | **OR** | **95% CI** | **p-value** |
| --- | --- | --- | --- |
| **Baseline KPS (continuous)** | **0.96** | **0.92-1.00** | **0.037** |
| Any smoking history (1=yes) | 1.64 | 0.69-3.86 | 0.260 |
| Superior constrictor mean dose (Gy) | 0.98 | 0.90-1.07 | 0.617 |
| Middle constrictor mean dose (Gy) | 1.04 | 0.99-1.10 | 0.126 |
| Oral cavity mean dose (Gy) | 1.05 | 1.00-1.10 | 0.074 |
| Lips mean dose (Gy) | 1.02 | 0.95-1.10 | 0.556 |
| Mandible mean dose (Gy) | 0.99 | 0.92-1.07 | 0.807 |
|  |  |  |  |
| *Multivariable logistic regression for persistent feeding-tube dependence at 6 months among patients with a gastrostomy who survived beyond 6 months and had complete predictor data (n = 188; 49 [26.1%] still dependent). OR > 1 indicates higher odds of remaining tube-dependent at 6 months. KPS, Karnofsky performance status; OR, odds ratio; CI, confidence interval.* | | | |

**Figure S1. Unadjusted landmark Kaplan–Meier analysis of overall survival by feeding-tube status (PEG/RIG cohort, n = 204)**

Patients alive at each landmark (3, 6 or 12 months after treatment start) were classified as tube-independent or still tube-dependent according to tube status known at that time point, and survival was measured from the landmark onward. Curves are unadjusted Kaplan–Meier estimates; hazard ratios are from univariable Cox regression. (A) 3-month landmark: 200 at risk, 106 still dependent; HR 1.71 (95% CI 0.98–3.00), p = 0.059. (B) 6-month landmark: 194 at risk, 51 still dependent; HR 1.78 (95% CI 1.01–3.13), p = 0.048. (C) 12-month landmark: 186 at risk, 27 still dependent; HR 2.53 (95% CI 1.30–4.91), p = 0.006. Covariate-adjusted curves and estimates are shown in Figure 3 and Table 4 of the main manuscript. HR, hazard ratio; CI, confidence interval.
